# Retraction: A well-defined S-g-C_3_N_4_/Cu–NiS heterojunction interface towards enhanced spatial charge separation with excellent photocatalytic ability: synergetic effect, kinetics, antibacterial activity, and mechanism insights

**DOI:** 10.1039/d6ra90074g

**Published:** 2026-07-24

**Authors:** Haya A. Abubshait, Shahid Iqbal, Samar A. Abubshait, Mohammed T. Alotaibi, Norah Alwadai, Nada Alfryyan, Hashem O. Alsaab, Nasser S. Awwad, Hala A. Ibrahium

**Affiliations:** a Basic Sciences Department, Deanship of Preparatory Year and Supporting Studies, Imam Abdulrahman Bin Faisal University P. O. Box 1982 Dammam 31441 Saudi Arabia; b Department of Chemistry, School of Natural Sciences (SNS), National University of Science and Technology (NUST) H-12 Islamabad 46000 Pakistan shahidiqbal.chem@sns.nust.edu.pk; c Department of Chemistry, College of Science, Imam Abdulrahman Bin Faisal University P. O. Box 1982 Dammam 31441 Saudi Arabia; d Department of Chemistry, Turabah University College, Taif University P. O. Box 11099 Taif 21944 Saudi Arabia; e Department of Physics, College of Sciences, Princess Nourah Bint Abdulrahman University P. O. Box 84428 Riyadh 11671 Saudi Arabia; f Department of Pharmaceutics and Pharmaceutical Technology, Taif University P. O. Box 11099 Taif 21944 Saudi Arabia; g Chemistry Department, Faculty of Science, King Khalid University P. O. Box 9004 Abha 61413 Saudi Arabia; h Biology Department, Faculty of Science, King Khalid University P. O. Box 9004 Abha 61413 Saudi Arabia; i Department of Semi Pilot Plant, Nuclear Materials Authority P. O. Box 530 El Maadi Egypt

## Abstract

Retraction of “A well-defined S-g-C_3_N_4_/Cu–NiS heterojunction interface towards enhanced spatial charge separation with excellent photocatalytic ability: synergetic effect, kinetics, antibacterial activity, and mechanism insights” by Haya A. Abubshait *et al.*, *RSC Adv.*, 2022, **12**, 3274–3286, https://doi.org/10.1039/D1RA07974C.

The Royal Society of Chemistry hereby wholly retracts this *RSC Advances* article due to concerns with the reliability of the data.

The degradation experiments in Fig. 6a–d were first published in ref. 1 and described as different experiments.

The transient photocurrent response data in Fig. 8d is partially identical to Fig. 8d in ref. 1.

The authors provided a response and new data but they did not address the concerns and they were not able to provide the original raw data.

Given the significance of these concerns, the findings presented in this paper are no longer reliable.

The authors did not respond to any correspondence regarding the retraction of this article. Haya A. Abubshait, Samar A. Abubshait, Hashem O. Alsaab and Nasser S. Awwad could not be contacted.

This retraction supersedes the information provided in the Expression of Concern related to this article.

Signed: Laura Fisher, Executive Editor, *RSC Advances*

Date: 6th July 2026
